# Trends in the utilisation of aged care services in Australia, 2008–2016

**DOI:** 10.1186/s12877-019-1209-9

**Published:** 2019-08-06

**Authors:** Jyoti Khadka, Catherine Lang, Julie Ratcliffe, Megan Corlis, Steve Wesselingh, Craig Whitehead, Maria Inacio

**Affiliations:** 1 Healthy Ageing Research Consortium, Registry of Older South Australians, South Australian Health and Medical Research Institute, North Terrace, PO Box: 11060, Adelaide, SA 5001 Australia; 2 Institute for Choice, University of South Australia, Adelaide, Australia; 3 College of Nursing and Health Sciences, Flinders University, Adelaide, Australia; 4 Helping Hand, Adelaide, Australia; 5 Division of Health Sciences, University of South Australia, Adelaide, Australia

**Keywords:** Aged care, Utilisation, Incidence rate, Home care, Permanent residential care, Respite care, Transition care

## Abstract

**Background:**

Aged care support services in Australia are delivered through home care packages, permanent residential care, respite care and transition care. This study aimed to determine age and gender specific incidence rates of aged care service utilisation in Australia between 2008-09 and 2015–16.

**Methods:**

This is a population-based epidmiological study of people accessing aged care services in Australia. The trends and characteristics of people (over the age of 65 years old) accessing aged care services in Australia were evaluated, using data (2008–09 and 2015–16) from the Australian Institute of Health and Welfare and Australian Bureau of Statistics. The yearly utilisation incidence rates (per 1000 people) per service type were calculated and changes in incidence rate ratios (IRR) of service utilisation for the study period were estimated using Poisson regression models.

**Results:**

The proportion of older Australians aged ≥65 years who used aged care services remained similar between 2008-09 (5.4%, *N* = 208,247) and 2015–16 (5.6%, *N* = 248,669). However, the incidence use of specific services changed during the study period. Specifically, admissions into permanent residential care decreased (from 23.8/1000 people in 2008–09 to 19.6/1000 in 2015–16, at a IRR of 0.84/year, *p* < 0.001) but increased for transition care (from 4.3/1000 in 2008–09 to 6.6/1000 in 2015–16, at a IRR of 1.57/year, *p* < 0.001) and home care packages (from 8.04/1000 in 2008–09 to 12.0/1000 per 1000 in 2015–16, at a IRR of 1.52/year, *p* < 0.001). Between 2008-09 and 2015–16, the greatest changes in IRR were observed in males aged 80–89 years accessing transition care (IRR = 1.68/year, *p* < 0.001). A higher proportion of people aged between 80-89 years (≥45%), females (≥60%), Australia born (≥ 60%) and English speakers (≥80%) used all the service types.

**Conclusions:**

Patterns of service utilisation for aged care services changed over the study period with a decrease in incidence of individuals accessing permanent residential care but increased for other service types. This finding reflects changes in attitudes regarding ageing in place and policies. These findings are helpful to inform key stakeholders on service planning to further improve quality of the aged-care services in Australia.

## Introduction

In common with other Organization for Economic Co-operation and Development countries, the population of Australia is progressively aging with a relatively larger proportion of its population aged 65 years or above compared to 20 years ago [1]. In 2015–16, 15% (3.4 million) of residents were over 65 years old and this proportion is projected to increase to 19% by 2031 [2]. Currently 7% of those over 65 years of age are receiving residential aged care services [3]. This group are also heavy users of health services accounting for a significant proportion (41%) of all hospitalisations and days spent in hospital (48%) [4]. In 2015–16 the Australian government spent $17 billion dollars on aged care services, with over two thirds of this spending allocated to residential aged care services, and this expenditure is expected to rise significantly over the coming decades [5].

**Fig. 1:**
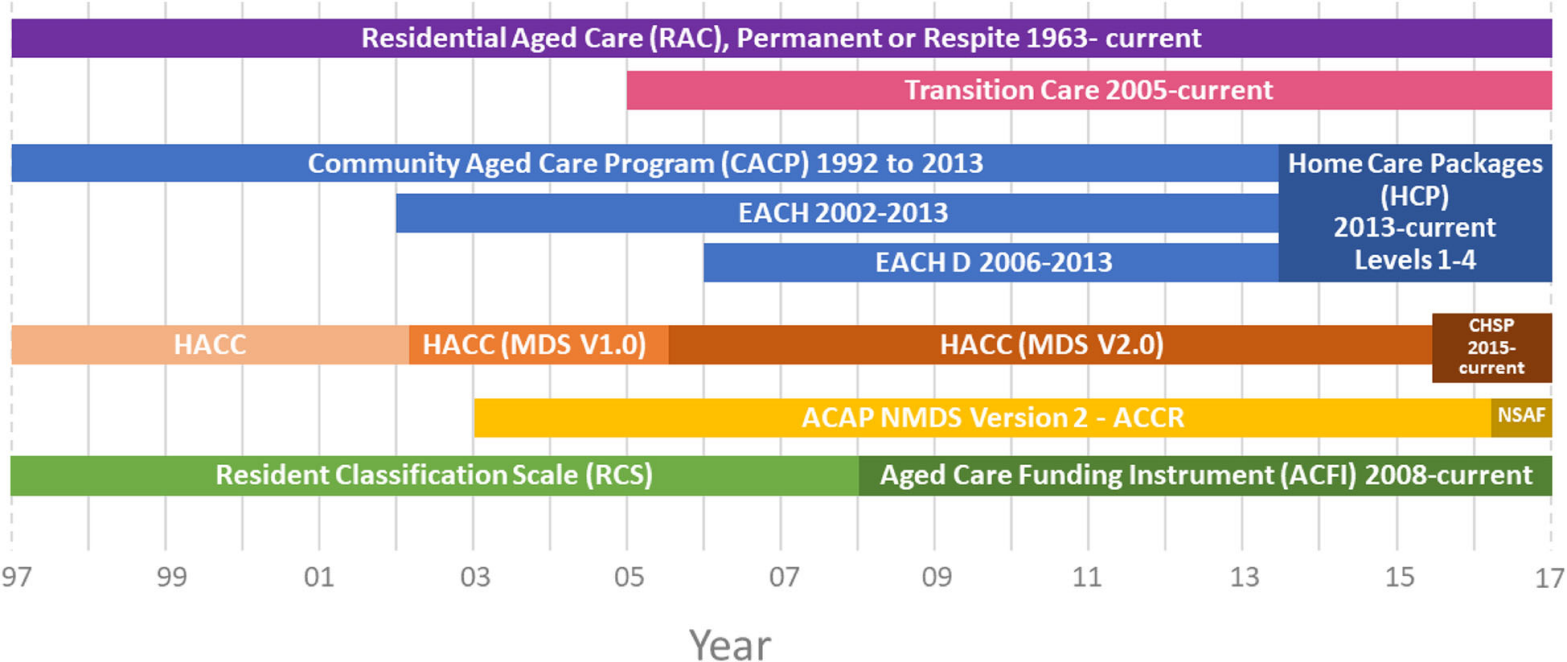
Timeline of Aged Care Programmes in Australia, 1997-current. Note: EACH = Extended Care at Home; EACH-D = Extended Care at Home-Dementia; HACC = Home and Community Care; MDS = Minimum Data Set; CHSP = Commonwealth Home Support Programme; ACAP = Aged Care Assessment Programme; NMDS = National Minimum Data Set; ACCR = Aged Care Client Record, NSAF = National Screening and Assessment Form

Aged care services in Australia have been subsidised by the Federal Government since 1963 and over time have evolved into a variety of service provision programmes. Commencing 2003 an Aged Care Assessment Team (ACAT) approval is required to access aged care services. An ACAT carries out assessments under the Federal Government’s Aged Care Assessment programme (ACAP) to determine an individual’s care needs, eligibility for services, recommendations for specific type of support needed and the level of aged care programme to be assigned. Briefly, aged care services currently in existence in Australia are:

1. *Home care packages (HCP):* HCP have a focus on supporting people to remain living at home and in their own communities for as long as possible and thereby preventing premature or inappropriate admission to permanent residential aged care. Both in 2013 and 2015, the structure of HCP changed, with programmes in existence previously (Community Aged Care Packages, Extended Aged-Care at Home; and Extended Aged Care at Home-Dementia, Home and Community Care) replaced by ‘Home Care Packages Programme’ and ‘Commonwealth Home Support Programme’ respectively (Fig. 1);
2. *Residential Aged Care (RAC):* There are two types of programmes delivered through RAC services: permanent residential aged care (PRAC) and respite residential care (RRC) [5]. *PRAC* provides government subsidised institutional residential aged care services for those who are not coping well at home. Government funding is allocated based on means-tested income fees for these services. *RRC* provides short-term relief or break to care recipients or their usual carers in residential care facilities from their usual arrangements. This may be arranged for planned breaks, holidays or in case of emergencies [6];
3. *Transition Care (TC):* TC provides a short-term care to older people leaving hospital or those who have accessed or who are deemed eligible for at least low-level PRAC. TC aims to provide services during the transition to facilitate recovery from illness, improve functioning and independence to delay or avoid entry into PRAC [7].

The aged care services are co-funded by both the Australian Government and the service users. The government regulates the maximum costs clients are required to pay and subsidises a range of services. The amount of government subsidy is determined by the types of services (e.g. home vs residential aged care), level of care needs and the individual’s income and/or assets (i.e. means-tested). The Australian Government also has targets for subsidised long-term care places for every 1000 people over 70 years old, which were around 111–113 during the study period, and the ratio of home care to residential care has been increasing [8].

As the aged care services and their provision in Australia have changed dramatically in the last couple of decades due to legislative reforms [9], changes in demographics and health care needs of the recipients [3, 10], and consumer preferences have also likely changed. Therefore, population based epidemiological evaluations of the change in incidence rate of aged care utilisation across different service types and subgroups of individuals would be of value to understand the changes in individuals accessing services. Specifically, the aim of this study was to describe patterns of service use and to estimate the rate of change by age and gender specific groups between 2008-09 and 2015–16 in Australia.

## Methods

### Study design, setting, and data sources

A population based epidemiological study was conducted using publicly available data from Australian Institute of Health and Welfare (AIHW) GEN Aged Care Data [11–13] and the Australian Bureau of Statistics (ABS). From the AIHW GEN Aged Care Data the de-identified datasets describing admissions into aged care were obtained. From the ABS Australian historical population estimates for the same time periods as the AIHW GEN Aged Care data were obtained.

### Study population

People ≥65 years who were admitted to HCP, RAC, and TC aged care programmes in Australia between July 1st 2008 to June 30th 2016 were included.

### Variables

The incidence of annual admissions into PRAC, RRC, HCP, or TC services were obtained from the AIHW GEN Aged Care data admissions files for the years of 2008–09 to 2015–16 [12]. These datasets offer de-identified information on new admissions into services each year, including the individuals’ age, gender, country of birth, preferred language spoken at home and indigenous status. We accessed the datasets between 2008-09 and 2015–16 to determine trends of admission into different aged care services.

Cohort characteristics evaluated by year included: age (stratified as 65–79, 80–89, ≥90 years); gender; country of birth (stratified as Australia, Other Non-English-speaking country, Other English-speaking country); preferred language spoken at home (English vs other).

### Statistical analysis

Data were analysed using SAS (Version 9.2, SAS Institute, Cary, NC, USA) and STATA MP 15.1 (StataCorp, Texas, USA). Summary statistics including frequencies and proportions were calculated to describe the study population. The incidence of aged care service utilisation rate and 95% confidence intervals (CI) per 1000 Australian citizens ≥65 years (from ABS data) per financial year were estimated. Overall incidence of aged care service utilisation rates and by age and gender groups were evaluated. Historical changes in the incidence of aged care utilisation per year were modelled using Poisson regression. The changes in the incidence of aged care service utilisation rate over time were calculated using incidence rate ratios (IRRs). Models were adjusted by age, gender, and state. IRRs and 95% CI were reported. All reported *P* values were considered statistically significant when less than 0.05 (α). This study adheres with the tenets of the declaration of Helsinki.

## Results

In 2015–16, there were 3.7 million (15% of total population) people age 65 years old and older, an increase of 0.79 million (27%) from 2008 to 09 in Australia. Over the same period, older people accessing aged care service increased by 19%, from 208,247 in 2008–09 to 248,669 in 2015–16. Both in 2008–09 and 2015–16, a higher proportion of older people entering the aged care system were females (> 60%), aged between 80-89 years old (≥45%), Australia born (> 63%), English speaking (> 80%) and non ATSI (> 98%), Table 1.

**Table 1:** Characteristics of people admitted to Aged Care Services in 2008–09 and 2015–16

	Home care				Residential care								Transition care			
	2008–09		2015–16		Permanent				Respite				2008–09		2015–16	
					2008–09		2015–16		2008–09		2015–16					
	N	%	N	%	N	%	N	%	N	%	N	%	N	%	N	%
All	23340	100.0	44074	100.0	69171	100	72126	100.0	56524	100	73335	100	12571	100.0	24270	100.0
Gender^a^																
Female	15478	66.3	27994	63.5	43337	62.7	43525	60.3	34642	61.3	43592	59.4	8127	64.6	14998	61.8
Male	7862	33.7	16050	36.4	25834	37.3	28601	39.7	21882	38.7	29743	40.6	4444	35.4	9272	38.2
Age group^a^																
65–79	7755	33.2	15408	35.0	18367	26.6	19006	26.4	16991	30.1	20329	27.7	4582	36.4	9532	39.3
80–89	11774	50.4	20809	47.2	36020	52.1	34595	48.0	28971	51.3	35119	47.9	6259	49.8	11226	46.3
≥ 90	3811	16.3	7853	17.8	14783	21.4	18525	25.7	10562	18.7	17887	24.4	1730	13.8	3512	14.5
Indigenous Status																
Aboriginal or Torres Strait Islander	530	2.3	587	1.3	412	0.6	699	1.0	573	1.0	976	1.3	125	1.0	220	0.9
Neither Aboriginal nor Torres Strait Islander	22797	97.7	22240	50.5	68651	99.2	71413	99.0	55950	99.0	72359	98.7	12446	99.0	24050	99.1
Unspecified	13	0.1	21247	48.2	108	0.2	14	< 0.1	1	< 0.1	0	0.0	0	0.0	0	0.0
Country of Birth																
Australia	15606	66.9	28046	63.6	49350	71.3	49616	68.8	40409	71.5	50343	68.6	8529	67.8	16461	67.8
Non-English- speaking countries	4588	19.7	9669	21.9	11044	16.0	13330	18.5	8751	15.5	13910	19.0	2403	19.1	4766	19.6
Other English- speaking countries	3047	13.1	5445	12.4	8478	12.3	8960	12.4	7210	12.8	8824	12.0	1580	12.6	2945	12.1
Not stated	99	0.4	914	2.1	299	0.4	220	0.3	154	0.3	258	0.4	59	0.5	98	0.4
Language																
English	20646	88.5	36034	81.8	62682	90.6	65514	90.8	51446	91.0	66321	90.4	11179	88.9	21931	90.4
Other languages	2651	11.4	5147	11.7	6406	9.3	6532	9.1	5016	8.9	6945	9.5	1371	10.9	2309	9.5
Not stated	43	0.2	2893	6.6	83	0.1	80	0.1	62	0.1	69	0.1	21	0.2	30	0.1
State																
Australian Capital Territory	439	1.9	829	1.9	751	1.1	1,155	1.6	936	1.7	737	1.0	220	1.8	332	1.4
New South Wales	8174	35.0	13890	31.5	24452	35.4	24718	34.3	22172	39.2	28830	39.3	4132	32.9	7636	31.5
Northern Territory	274	1.2	545	1.2	198	0.3	170	0.2	357	0.6	396	0.5	86	0.7	121	0.5
Queensland	4332	18.6	9628	21.8	11938	17.3	13197	18.3	7394	13.1	9332	12.7	2044	16.3	4850	20.0
South Australia	1883	8.1	3631	8.2	6234	9.0	6189	8.6	5335	9.4	7879	10.7	1202	9.6	2050	8.4
Tasmania	538	2.3	998	2.3	1852	2.7	2032	2.8	1939	3.4	2188	3.0	340	2.7	595	2.5
Victoria	5234	22.4	10109	22.9	17855	25.8	18825	26.1	14494	25.6	19945	27.2	3729	29.7	6529	26.9
Western Australia	2466	10.6	4440	10.1	5835	8.4	5840	8.1	3848	6.8	4028	5.5	818	6.5	2157	8.9

^a^In the home care group there are 30 (< 0.1%) individuals with unspecified gender and 4 (< 0.1%) with unspecified age in 2015–16. In the permanent residential care group there is 1 (< 0.1%) with unspecified age in 2008–09. In the transition group, there is 125 (1%) with unspecified age in 2008–09 and 220 (0.9%) with unspecified age in 2015–16

**Fig. 2:**
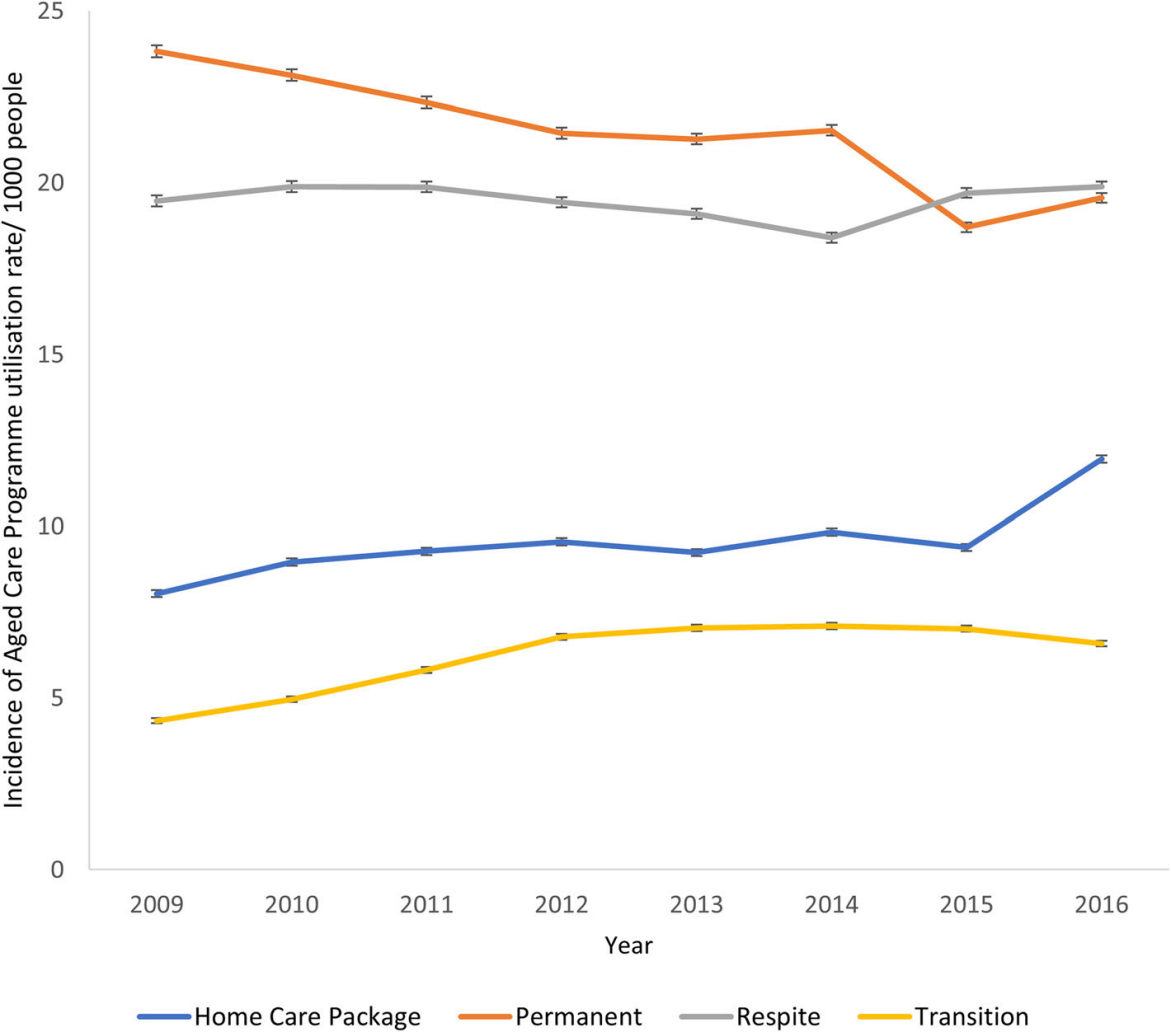
Incidence of Aged Care Programme Utilisation Rate/1000 people 65 Years Old and Older in Australia, 2008–09 to 2015–16

The proportion of Australian residents age 65 years old and older who used the aged care services remained the same between 2008-09 (5.4%, *N* = 208,247) and 2015–16 (5.6%, 248,669). However, the incidence rate of admission to specific types of services changed during the study period, Table 1. The incidence rate of admissions to PRAC decreased from 23.8/1000 people in 2008–09 to 19.6/1000 in 2015–16, at an adjusted IRR of 0.84/year (*p* < 0.001) over the period. The incidence rate of admission to HCP services increased from 8.04/1000 in 2008–09 to 12.0/1000 in 2015–16 at a IRR of 1.52/year (*p* < 0.001) over the period and for TC services from 4.3/1000 in 2008–09 to 6.6/1000 in 2015–16 at a IRR of 1.57/year (*p* < 0.001), Fig. 2. The incidence rate of admission to respite care increased slightly from 19.5/1000 in 2008–09 to 19.9/1000 in 2015–16, at a IRR of 1.05/year (*p* < 0.001) over the study period.

**Fig. 3:**
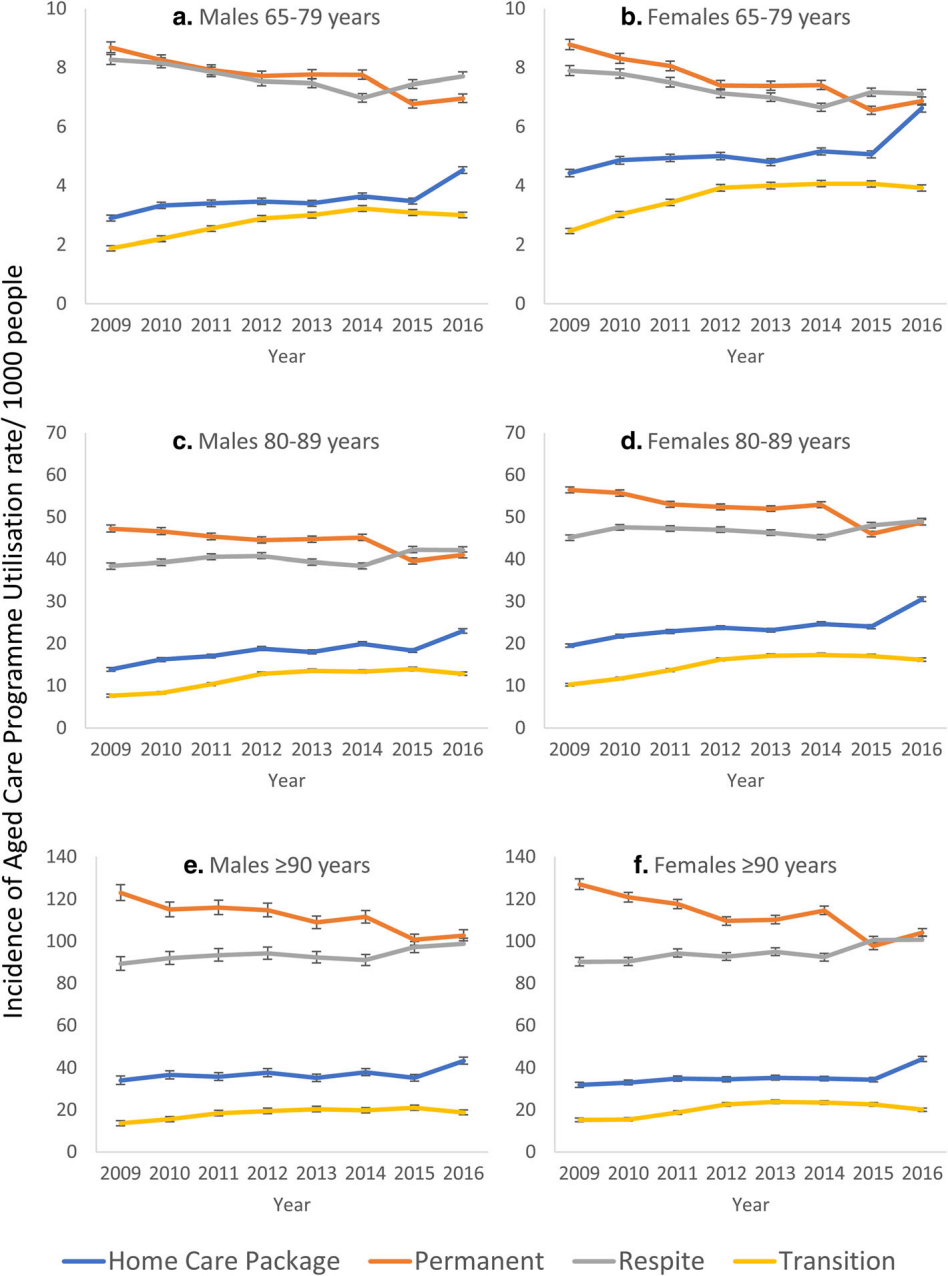
Incidence of Aged Care Programme Utilisation Rate/1000 people 65 Years Old and Older in Australia by Age and Gender, 2008–09 to 2015–16

Within specific gender and age groups, the highest changes in IRRs of admission to aged care services during the study period were observed in 80–89 years old males accessing TC, from 11.0/1000 in 2008–09 to 17.4/1000 in 2015–16, at a IRR of 1.68/year (*p* < 0.001), and HCP from 24.3/1000 in 2008–09 to 37.9/1000 in 2015–16 at a IRR of 1.65/year (*p* < 0.001), Table 2. The highest reduction in IRRs of admission to aged care services was observed in 65–79 years old females accessing PRAC, from 8.6/1000 in 2008–09 to 6.8/1000 in 2015–16, at a IRR of 0.78/year (*p* < 0.001). The incidence rate of admission to respite care also decreased in 65–79-year-old females, from 8.9/1000 in 2008–09 to 8.0/1000 in 2015–16 at a IRR of 0.91/year (*p* < 0.001), and in 65–79 years old males from 8.8/1000 in 2008–09 to 8.2/1000 in 2015–16 at a IRR of 0.93/year (*p* < 0.001), whereas all other gender and age groups observed increases in incidence rates of admissions (Table 2 & Fig. 3). Between 2014 and 2015, in those aged 79 year and above a decrease in incidence rate of admission to PRAC and increase in admission to HCP and respite care services was observed, Fig. 3.

**Table 2:** Crude and adjusted incidence rate ratio of change in Aged Care Programme Utilisation between 2008-09 and 2015–16, overall and by gender and age groups

	Home care		Residential care				Transition Care	
			Permanent		Respite			
	Crude IRR (95%CI)	Adjusted IRR (95%CI)^1^	Crude IRR (95%CI)	Adjusted IRR (95%CI)^1^	Crude IRR (95%CI)	Adjusted IRR (95%CI)^1^	Crude IRR (95%CI)	Adjusted IRR (95%CI)^1^
Overall	1.49 (1.46–1.51)	1.52 (1.50–1.55)	0.82 (0.81–0.83)	0.84 (0.83–0.85)	1.02 (1.01–1.-03)	1.05 (1.03–1.06)	1.52 (1.49–1.55)	1.57 (1.53–1.60)
Females 65–79	1.50 (1.45–1.55)	1.49 (1.44–1.45)	0.78 (0.76–0.81)	0.78 (0.76–0.81)	0.90 (0.88–0.93)	0.91 (0.88–0.93)	1.59 (1.52–1.67)	1.59 (1.52–1.67)
Females 80–89	1.56 (1.52–1.61)	1.59 (1.52–1.60)	0.86 (0.85–0.88)	0.86 (0.85–0.88)	1.09 (1.07–1.11)	1.09 (1.07–1.11)	1.58 (1.52–1.65)	1.58 (1.52–1.64)
Females ≥ 90	1.39 (1.32–1.45)	1.39 (1.32–1.45)	0.82 (0.80–0.84)	0.82 (0.80–0.84)	1.12 (1.09–1.15)	1.12 (1.09–1.15)	1.32 (1.24–1.42)	1.32 (1.23–1.41)
Males 65–79	1.56 (1.50–1.63)	1.56 (1.49–1.63)	0.80 (0.78–0.83)	0.80 (0.78–0.83)	0.93 (0.90–0.96)	0.93 (0.91–0.96)	1.59 (1.51–1.68)	1.59 (1.51–1.68)
Males 80–89	1.28 (1.19–1.37)	1.65 (1.59–1.72)	0.87 (0.83–0.89)	0.87 (0.85–0.89)	1.10 (1.07–1.13)	1.10 (1.07–1.13)	1.68 (1.60–1.77)	1.68 (1.60–1.77)
Males ≥ 90	1.49 (1.46–1.51)	1.28 (1.19–1.37)	0.84 (0.80 = 0.87)	0.84 (0.80–0.87)	1.11 (1.06–1.16)	1.10 (1.06–1.15)	1.38 (1.23–1.54)	1.38 1.23–1.54)

*IRR* Incidence rate ratio, *CI* Confidence Intervals

^1^Overall estimates were adjusted by age, gender and state. Others adjusted by age and state. All *P* values were < 0.001

## Discussion

This study provides a comprehensive Australia wide incidence of admission into services, rate of change in admissions, and demographic profiles of older people who have commenced aged care services during the most recent period for which data is available. The proportion of people accessing aged care services remained similar over the study period despite significant growth (by 27%) of the general population aged older than 65 years old in Australia. However, the trends of admissions into services changed during this period with a significant decrease in the uptake of PRAC coinciding with increases in other services, with the highest increase observed in the admission to HCP. A noticeable growth in HCP admission was observed 2014 onwards, which aligns with the introduction of a new Home Care Packages Programme by the Federal government [14].

Over the study period, the incidence rate of admission into PRAC services declined gradually until 2014 and since then a noticeable decline in admissions into PRAC was observed. This is evident in both genders and all age groups. Similar recent declines in institutionalised long-term care services utilisation and occupancy rates have also been reported in other countries, including Germany, Netherlands and United States [15, 16]. The decline in admission rates into PRAC in Australia has coincided with a consequent increase in the utilisation of HCP services. This is likely the result of significant policy changes in the sector placing more emphasis on home and community care services and encouraging the transition to PRAC only when other service options are exhausted and shifting targets by the Australian Government regarding age care operational provision rate for HCP and PRAC in the last few years [8]. Despite population ageing, the shift observed in this study to aging in place is also occurring in most developed countries [17]. The attractiveness of community living could be attributed to several reasons such as the wish of older people to stay in their own homes and communities and thus maintain their autonomy, increase in privately funded assisted living (e.g. retirement villages), availability of better health and primary care services that maintain individuals at home (e.g. better chronic disease management, access to flu vaccines, reduced smoking rates) [18, 19].

Andres et al. also argued that government policies could directly influence people’s preference of home care over institutional care [15]. A noticeable change in pattern of utilisation of home and permanent care was observed after the Australian government introduced several reforms such as a web based portal called “My Aged Care,” merging of three community based programmes (Community Aged Care Programme, Extended Aged Care at Home, Extended Aged Care at Home-Dementia) into a new HCP programme, greater investment in HCP programmes and increasing in target ratios of HCP to PRAC places [8], and launching of the Aged Care Pricing Commission [20]. The short-term effects in the patterns of utilisation could be attributed to these government reforms. Since 2013, Australian government has announced additional reforms to be implemented progressively over 10 years. As observed in this study, the utilisation of aged care services is intrinsically linked to policy change, therefore caution is needed to avoid the realisation of unwanted consequences of new policies, including older frail people who are considered eligible not being able to obtain necessary services or having long waits to enter the service due to funding or reduced placement levels.

English speaking, females, 80–89 years old constituted a large segment of our cohort and has been consistent over the years evaluated. Borotkanics et al. [21], and Forder et al. have reported that females aged between 70 and 90 years are more likely to enter PRAC [22]. Increased longevity is due to better health and primary health care, and so it is not surprising that aged care services are being used more often by people who have lived to a very advanced age which was identified in the study. Very old individuals are the ones who need supportive care services as they are nearing the end of life and this is also at a time of life when spousal death and the death of others who could be their first line of care [23]. We also found that a lower proportion of people entering PRAC were born in non-English speaking countries (< 20%) and had a preferred language (< 12%) other than English (Culturally And Linguistically Diverse, CALD), which had been reported by AIHW for the national cohort and studies by Petrov et al. and Jorgensen et al. [4, 11, 24]. When compared to Australian general population (about 21% spoke a language other than English), the proportion of older CALD people using PRAC was low [25]. The study has highlighted the under-representation of older people from ATSI and CALD population groups accessing aged care services. Despite ATSI people accounting for almost 5% of Australians ≥65 years, the difference in utilisation of the aged care services in this study was substantial between ATSI (< 1% for all except for HCP) and non-Indigenous people (> 97%) and this remained consistent over the study period [21, 26]. LoGiudice et al. have highlighted the importance of developing a culturally appropriate care system that preserve ATSI identity, staffing, policy, planning and resourcing are necessary to cater the needs of the older ATSI population [27]. HCP utilisation rates was relatively higher over the study period in this demographic, which may indicate that the older ATSI population were more likely to opt to remain for as long as possible at home and in the community rather than accessing residential facilities. To date very few studies have explored the issue of aged care services inequity for the older ATSI population [27–29]. Further research is needed studies to explore the support needs and preferences of the older ATSI population and incorporate this evidence into service innovations to assist in increasing the uptake of aged care services in this population.

The main limitation of this study includes the reliance on publicly available data, which has limited variables (e.g. type of facility or service provider, overall health and frailty status of residents) to explore the factors that potentially influence the changes observed. Therefore, this study necessarily provides a limited evaluation of factors that influenced the changes in use of aged care services, rather than an exhaustive exploration of the possible factors. However, we believe that the influence of the variables evaluated in this study are likely significant in the trends of aged care use and combined with the external factors discussed (i.e. recent aged care reforms) accurately depict the main changes during the study period. The incidence of aged care service utilisation presented in these analyses are also estimates, which are limited because publicly available census data include random adjustments to small cells for privacy reasons, which introduces an additional small margin of error (i.e. wider confidence intervals). Contrary, our estimates rates could suffer from potentially lower variability estimates (i.e. smaller confidence intervals) because of our inability to account for potential multiple entries into the same service by the same person as our data does not include individual identifiers. While it is unlikely that this would occur in those accessing home care, permanent residential aged care, and transition care, it is possible for the respite care estimates. Finally, the incidence rates of service admission were calculated based on the proportion of the Australian population aged 65 years old and older, but not all Australians users of aged care services were included in this evaluation. For example, indigenous people may access age care services when they are 50 years of older and under certain circumstances younger people with disabilities (50–65 years ATSI are < 0.3% of the total population receiving aged care). However, as the vast majority of aged care recipients are aged 65 years and over and the main focus of this study.

## Conclusions

The provision of aged care services in Australia is complex and diverse but also dynamic due to the constant introduction of government reforms, and some changes in demographics and the way people use aged care services. Our findings indicate that although the numbers of people getting aged care services has increased with population growth and aging, the proportion of older people who are needing and getting supportive aged care services has not changed in the decade studied. Furthermore, there was a clear shift to home care services from institutionalised care and also there has been an aging trend among the people who are accessing aged care services with more older people accessing services than younger people. Having home care services readily available could be a key factor not only in keeping people out of nursing homes but also keeping them well and out of hospital. With the current emphasis on the government’s “age in place” policy, understanding the trends in aged care service utilisation is helpful to key stakeholders in informing service planning and the future allocation of funding and resources.

## Data Availability

The data that support the findings of this study are openly available from the Australian Institute of Health and Welfare (AIHW) GEN Aged Care Data (Source: https://www.gen-agedcaredata.gov.au/Resources/Access-data/2017/August/GEN-Data-People-using-aged-care and https://www.gen-agedcaredata.gov.au/Resources/Access-data/2018/June/GEN-data-Admissions-into-aged-care) [11, 12] and the Australian Bureau of Statistics (ABS), http://www.abs.gov.au/ausstats/abs@.nsf/mf/3101.0/ [13]

## Abbreviations

ABS: Australian Bureau of Statistics
ACAT: Aged Care Assessment Team
AIHW: Australian Institute of Health and Welfare
ATSI: Aboriginal and Torres Strait Islander
CACP: Aged Care Packages
CALD: Culturally and Linguistically Diverse
CI: Confidence intervals
EACH: Extended Aged-Care at Home
EACHD: Extended aged-care at home-dementia
HACC: Home and Community Care
HCP: Home care package(s)
IRR: Incidence rate ratios
PRAC: Permanent residential aged care
RAC: Residential aged care
RRC: Respite residential care
TC: Transition care
